# Association between maternal postpartum depression and children's physical growth in early childhood: a birth cohort study

**DOI:** 10.3389/fped.2023.1135876

**Published:** 2023-07-26

**Authors:** Qiong He, Gang Cheng, Simin He, Gang Tian, Xiaowei Xie, Ni Jiang, Xianying Min, Chao Li, Rui Li, Yan Shi, Tong Zhou, Yan Yan

**Affiliations:** ^1^ Department of Epidemiology and Health Statistics, Xiangya School of Public Health, Central South University, Changsha, China; ^2^ Medical Record Management and Statistical Information Center, Xiangya Hospital, Central South University, Changsha, China

**Keywords:** postpartum depression, physical development, birth cohort, children, panel data

## Abstract

**Background:**

Untreated maternal postpartum depression (PPD) has consequences for children's physical growth, but no published study has evaluated changes in this effect over time. Here we therefore aimed to evaluate the dynamic effects of PPD on the physical growth of children in a prospective birth cohort.

**Methods:**

Between 2015 and 2019, 960 mother-child pairs in Changsha, China were followed up when the child was aged 1–48 months. Data were obtained through household surveys. The mothers' depressive symptoms were measured using the Edinburgh Postpartum Depression Scale (EPDS) at 1 month postpartum. Linear mixed models were used to examine the changes in the association of PPD and EPDS scores with physical growth in six different age groups of children between 1 and 48 months.

**Results:**

A total of 604 mother-child pairs completed the follow-up, and 3.3% of mothers reported PPD. No associations were found between PPD and weight or height growth at any age. While EPDS scores were associated with weight gain (*β *= −0.014, 95% CI (−0.025, −0.002), *P* = 0.024) and height growth (*β *= −0.044, 95% CI (−0.084, −0.004), *P* = 0.030) rates at 1–3 months, no associations were found in older children.

**Limitations:**

The number of mothers who reported PPD was relatively small, and the measurement of PPD was not continuously taken.

**Conclusions:**

After adjustments for confounders, no dynamic association was found between PPD and children's weight and height growth. EPDS scores, in contrast, did negatively affect children's weight and height growth at age 1–3 months, but this effect was not long-lasting.

## 1. Introduction

Maternal postpartum depression (PPD) is considered a major depressive disorder that usually occurs within 4 to 6 weeks after childbirth, the reported worldwide prevalence is 7%–25% (1–3). Previous studies have reported that women have a greater risk of experiencing depression during the postpartum period (4) and that untreated PPD has consequences for the physical growth of children that extend beyond the normal screening period for depression: the children may be underweight, overweight, or shorter than expected (5–16). While it remains unclear how maternal PPD affects the physical growth of children, previous studies were based on the following hypotheses.

First, PPD may affect maternal rearing behavior and quality as well as the quantity and quality of interactions between mothers and children, thus resulting in changes in children's physical growth and development (9, 17, 18). For example, PPD may impair the ability of mothers to provide adequate nutrition to their children, resulting in underweight children in low-income countries (19, 20); in developed countries with obesity-causing environments, PPD may allow mothers to provide high-calorie fast and convenient food, leading to overweight children (21). Moreover, because of their focus on their emotions, depressed mothers have a poorer ability to interpret and respond to their children's signals in mother-child interactions compared with non-depressed mothers (22, 23). According to attachment theory, these early and unhealthy parent-child interaction problems will prevent children from establishing a sense of security with their mother, hinder their exploration of the external environment, and delay their motor and physical growth (19, 24). Second, the effects of PPD on children's physical development may vary with time and the duration of PPD; these effects are not limited to infancy, but also extend to early childhood, preschool-age, school-age, adolescence, and even adulthood (25, 26). For example, one study reported that chronic PPD lasting for 1 year was associated with low psychomotor and cognitive development in infants (27).

To date, many epidemiological studies have examined the association between maternal PPD and children's early physical growth, and some have suggested variations with income level. PPD seems to stunt children's physical growth in India (12, 13), Bangladesh (14), Zambia (15), Nigeria (5, 7), and other populations with low-income status (8). While, in Europe (6, 11, 16, 28, 29), in the United States (9, 10), and other high-income countries, PPD appears to have a positive association or no association with children's physical growth. However, no previous studies have reported dynamic associations between PPD and children's early physical growth, and potential changes in the effect of PPD on development 1-month postpartum or later have thus been ignored. Moreover, only a few studies included two (5, 9, 14) or more (8) measurements of children's physical development.

Dynamic evaluation of the association between maternal PPD and children's early physical growth can not only reveal the duration of the impact of PPD on children's physical growth but also reflect changes in this association at different stages. A dynamic view allows for the rapid identification of abnormal growth as a result of PPD and the implementation of necessary intervention measures. Recent studies have reported that many problems faced in adulthood, such as obesity, growth retardation, cardiovascular disease, mental health problems, and crime can be traced back to early childhood (30–32). Early childhood, especially infancy, is thus a crucial period affecting not only later childhood but one's entire life. Moreover, the effects of maternal PPD on offspring are not limited to the screening time for depression. In 2019, about 1,025,500 to 3,662,500 new mothers with 14.65 million newborns likely experienced depression in China (33), based on the global prevalence of PPD. Prospective studies that explore the association between PPD in mothers and offspring growth are therefore needed. Here we evaluated the dynamic effects of maternal postpartum depression on the early physical growth of children in a prospective birth cohort.

## 2. Methods

### 2.1. Study participants

A prospective ongoing birth cohort study including the streets of Sifangping, Dongfeng Road, and Xinhe in the Kaifu District of Changsha City, Hunan Province, China, was initiated in 2015. All participants are mothers living in these three streets and their children born between January and December 2015, all of whom were 48 months old at the end of 2019. The inclusion criteria were as follows: (1) mothers and children registered as permanent residents in the Kaifu District of Changsha City who had complete health records at the local community health service centers; (2) children with no history of mental illness or neurological disease; and (3) mothers who cooperated with the long-term follow-up and signed an informed consent form. The exclusion criteria were as follows: (1) mothers with severe neuropsychiatric or organic diseases and (2) mothers who did not complete the postpartum depressive symptom scale. All study procedures were in accordance with the ethical standards of the Independent Ethics Committee of Clinical Pharmacology Institute, Central South University, Changsha, China (CTXY-130041-3-2).

### 2.2. Measures

#### 2.2.1. Postpartum depression

Mothers' depressive symptoms were measured using the Chinese version of the Edinburgh Postpartum Depression Scale (EPDS) 1 month after childbirth (34, 35). The EPDS has 10 items, including mood (“I have been able to laugh and see the funny side of things”), fun (“I have looked forward with enjoyment to things”), self-blame (“I have blamed myself unnecessarily when things went wrong”), anxiety (“I have been anxious or worried for no good reason”), fear (“I have felt scared or panicky for no very good reason”), coping ability (“Things have been getting on top of me”), insomnia (“I have been so unhappy that I have had difficulty sleeping”), sadness (“I have felt sad or miserable”), crying (“I have been so unhappy that I have been crying”), and self-injury (“The thought of harming myself has occurred to me”), each of which was rated on a 4-point scale from 0 to 3 depending on the perceived severity of the symptom, giving a possible range of EPDS scores from 0 to 30 (34). We used a cut-off score of 13 to indicate PPD (34), analysing this as a categorical variable. We also analysed total EPDS score as a continuous variable.

#### 2.2.2. Children's physical growth

The physical growth indices of interest were children's weight and height at 1, 3, 6, 8, 12, 18, 24, 36, 42, and 48 months. These indices were obtained through household surveys conducted by the children's doctors.

#### 2.2.3. Covariates

Variables possibly linked to maternal postpartum depression or children's weight and height were chosen as covariates (36, 37), including:

(1) Child covariates: child sex (male or female), gestational week (<37 or ≥37 weeks), birth weight (<2,500 or ≥2,500 g), exclusive breastfeeding from birth to 1 month (yes or no), and disease from birth to 1 month (yes or no). A birth weight below 2,500 g was defined as low birth weight.
(2) Family covariates: maternal pre-pregnancy body mass index (BMI; <18.5, 18.5–23.9, ≥24 kg/m^2^), history of gestation (primiparous or multiparous), pregnancy disease (yes or no), age (<35 or ≥35 years old), education (senior high school or below, or bachelor's degree or above), paternal education (senior high school or below, or bachelor's degree or above), and average monthly household income (≤2,000 or >2,000 RMB). A pre-pregnancy BMI (BMI = weight [kg]/height [m]^2^) above 24 was defined as overweight and one below 18.5 was defined as thin.

### 2.3. Sample size

Given the probable persistence of the effect of PPD on children's physical development, we hypothesized that the association between PPD and children's weight or height would be negative and vary with children's growth. We tested this hypothesis using a prospective birth cohort study and used linear mixed models (LMMs) with repeated measurements to examine the association. Thus, the sample size of this study was estimated based on the formula $\left(m=\frac{{\left(Z_{1\text{-}\alpha /2}+Z_{1\text{-}\beta }\right)}^{2}\sigma^{2}(1+(n\text{-}1)\rho )}{\pi_{0}\pi_{1}\varphi_{1}^{2}n}\right)$ proposed by Liu and Liang (38) for repeated measurement data in a mixed linear model study. In this formula, *π*_1_ represents the prevalence of maternal PPD, *π*_0 _= 1–*π*_1_, *n* is the number of repeated measurements, *ρ* represents the correlation coefficient between the two measurements of the child's weight or height, $\varphi_{1}$ represents the slope of the change in the child's weight or height with the maternal PPD, and *σ*^2^ is the standard deviation of the residual. We assumed that *π*_1 _= 7% (1–3), *α* = 0.05, *β* = 0.10, *ρ* = 0.5, *σ*^2 ^= 1, and $\varphi_{1}=0.5$, and the number of repeated measurements was 10, resulting in a sample size required to show differences between groups of 355, at a significance level of 0.05 and a statistical power of 0.9. Considering the long follow-up period, we assumed a loss to follow-up rate of 20%, and therefore added 71 participants to the original calculated sample; the total required number of participants was thus 426. Our final sample consisted of 604 participants and thus met the minimum sample size requirement.

### 2.4. Statistical analysis

Categorical variables are presented as frequencies and percentages, and continuous variables as means and standard deviations. Chi-square tests and logistic regression analysis were used to compare differences in each child and family variable between the PPD and the non-PPD group. Repeated measures ANOVA was used to explore the growth patterns of children's weight and height in different months and to determine the interactions of PPD and covariates with time and their effect on children's weight and height.

LMMs were used to examine the dynamic association between PPD and children's weight and height at different periods between 1 and 48 months, at 1–3 months, 3–6 months, 6–12 months, 12–24 months, 24–36 months, and 36–48 months. PPD and EPDS scores were included in the models as double independent variables to examine the effect of maternal PPD on child development in a multidimensional way, and children's weight and height were included as the double dependent variable, alongside potential confounding family and child variables. In addition, the interactions of PPD, postpartum EPDS scores, or covariates with time found in the repeated measures ANOVA were also introduced into the LMMs. Estimates of regression coefficients (*β*) and corresponding 95% confidence intervals (CIs) were used to quantify the effects of PPD in the LMMs. A two-tailed *P-*value of less than 0.05 was considered to indicate statistical significance. All statistical analyses were performed using IBM SPSS 22.

## 3. Results

### 3.1. Participant characteristics

**Figure 1:**
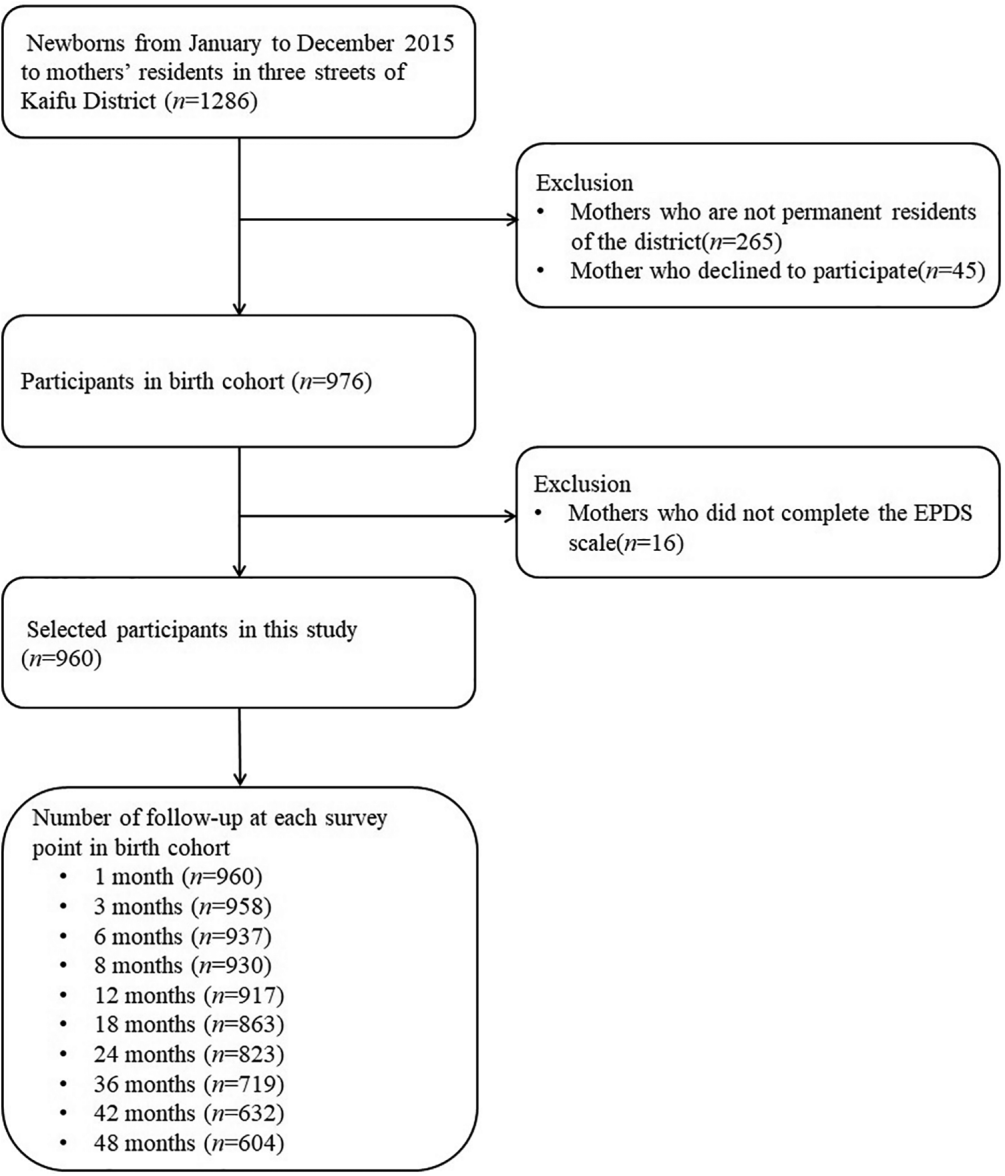
Study flow chart.

A flow chart of the participants in this study is shown in Figure 1. After excluding mothers who did not live in the study area, those who had no health care records (*n* = 265), declined to participate (*n* = 45), or did not complete the postpartum EPDS scale (*n* = 16), 960 mother-child pairs were included, 604 of which were finally followed up until the child reached the age of 48 months. The cumulative number of losses to follow-up in 4 years thus amounted to 356, resulting in a loss rate of 37.1%.

Analysis of the difference between the included and lost samples using the baseline data showed that the two samples were balanced in terms of maternal age (*χ*^2 ^= 3.68, *P* = 0.055), PPD (*χ*^2 ^= 0.47, *P* = 0.493), maternal education (*χ*^2 ^= 2.01, *P* = 0.156), parental education (*χ*^2 ^= 0.94, *P* = 0.331), average monthly household income (*χ*^2 ^= 0.80, *P* = 0.371), child sex (*χ*^2 ^= 2.23, *P* = 0.135), and proportion of children with low birth weight (*χ*^2 ^= 0.02, *P* = 0.883).

A summary of the 604 participant pairs' characteristics is provided in Table 1. Of the included children, 299 were boys (49.5%), 305 were girls (50.5%), and 18 (3.0%) had a low birth weight. Of the mothers, 20 had “PPD” (3.3%), 584 had “no PPD” (96.7%), and 481 (80.3%) exclusively breastfed their children during the 1-month postpartum period. A chi-square analysis showed that PPD were reported more frequently by mothers who were thin before pregnancy (40.0% vs. 16.4%, *P* = 0.012), those who were sick during pregnancy (45.0% vs. 13.3%, *P* = 0.001), those who had a lower average monthly household income (20.0% vs. 3.4%, *P* = 0.006), and those who had low birth weight babies (15.0% vs. 2.6%, *P* = 0.019), whereas PPD were reported less frequently by mothers who gave birth for the first time (15.0% vs. 49.3%, *P* = 0.003), and those who exclusively breastfed their children in the first month after delivery (50.0% vs. 81.3%, *P* = 0.002).

**Table 1:** Sample characteristics categorized by maternal postpartum depression.

Characteristics	Total (*N* = 604)	PPD (*N* = 20)	No PPD (*N* = 584)	*P*-value
Child characteristics				
Gender				0.617
Male	299	11 (45.0%)	288 (49.3%)	
Female	305	9 (55.0%)	296 (50.7%)	
Birth weight (g)				0.019*
<2,500	18	3 (15.0%)	15 (2.6%)	
≥2,500	582	17 (85.0%)	565 (97.4%)	
Exclusive breastfeeding from birth to 1 month				0.002*
Yes	481	10 (50.0%)	471 (81.3%)	
No	118	10 (50.0%)	108 (18.7%)	
Disease from birth to 1 month				0.011*
Yes	43	5 (25.0%)	38 (6.6%)	
No	554	15 (75.0%)	539 (93.4%)	
Family characteristics				
Maternal pre-pregnancy BMI (kg/m^2^)^#^				0.021*
<18.5	98	8 (40.0%)	90 (16.4%)	
18.5–23.9	374	11 (55.0%)	363 (66.2%)	
≥24	96	1 (5.0%)	95 (17.4%)	
Maternal history of gestation				0.003*
Primiparous	291	3 (15.0%)	288 (49.3%)	
Multiparous	313	17 (85.0%)	296 (50.7%)	
Maternal pregnancy disease				0.001*
Yes	86	9 (45.0%)	77 (13.3%)	
No	512	11 (55.0%)	501 (86.7%)	
Maternal Age (years)				
>35	538	19 (95.0%)	519 (88.9%)	0.713
≥35	66	1 (5.0%)	65 (11.1%)	
Maternal education				1.000
Senior high school or below	87	3 (15.0%)	84 (14.5%)	
Bachelor degree or above	514	17 (85.0%)	497 (85.5%)	
Paternal education				0.181
Senior high school or below	84	5 (25.0%)	79 (13.7%)	
Bachelor degree or above	514	15 (75.0%)	499 (86.3%)	
Average monthly household income (RMB)				0.006*
≤2,000	24	4 (20.0%)	20 (3.4%)	
>2,000	577	16 (80.0%)	561 (96.6%)	

Values are *n* (%). Due to the missing value of some variables, the total number of cases in subgroups was slightly different from the total number of cases. PPD, postpartum depression.

* Level of significance: *P* < 0.05.

^#^ A two-way comparison of PPD reporting between mothers in different pre-pregnancy BMI groups showed that pre-pregnancy thin mothers reported higher rates of PPD (*P* = 0.012).

However, multivariate logistic regression analysis adjusted for confounders showed that mothers who were thin before pregnancy (*OR* = 3.32, 95% CI (1.22–8.98), *P* = 0.018), those who were sick during pregnancy (*OR* = 3.37, 95% CI (1.27–8.99), *P* = 0.015), those who had a low monthly household income (*OR* = 5.05, 95% CI (1.37–18.59), *P* = 0.015) and those who did not exclusively breastfeed their children in the first month postpartum (*OR* = 3.93, 95% CI (1.52–10.18), *P* = 0.005) were at higher risk of reporting PPD.

### 3.2. Association between PPD and children's physical growth

The results of the repeated measures ANOVA showed that children's weight (*F* = 51881.20, *P* < 0.001) and height (*F* = 218869.20, *P* < 0.001) were increased linearly with age (indicated by the largest type III square and mean square), with differences in child weight (*β *= −0.282, 95% CI (−0.525, −0.038), *P* = 0.023) and height (*β *= −0.870, 95% CI (−1.650, −0.090), *P* = 0.029) between depressed and non-depressed mothers evident over the entire 48 months before adjustment. We therefore used the linear model as the basic mixed model to fit the growth trajectory of children's physical development.

In the unadjusted models, PPD were associated with both weight gain and height growth rates in children aged 1–3 months (*β *= −0.358, 95% CI (−0.612, −0.104), *P* = 0.006; *β *= −1.162, 95% CI (−2.002, −0.321), *P* = 0.007) (Table 2). However, after controlling for covariates, no association between PPD and children's weight and height growth was observed in either age model from1 to 48 months.

**Table 2:** Association between postpartum depression and children's physical growth at 1–48 months old.

Models (No PPD group as control)	Unadjusted				Adjusted			
	*β*	95% CI	*t*	*P*-value	*β*	95% CI	*t*	*P*-value
Weight models								
Model (1 to 3 months)	−0.358	(−0.612, −0.104)	−2.77	0.006*	−0.141	(−0.373, 0.090)	−1.20	0.231
Model (3 to 6 months)	−0.084	(−0.431, 0.263)	−0.48	0.633	0.069	(−0.261, 0.398)	0.41	0.682
Model (6 to 12 months)	0.055	(−0.335, 0.444)	0.28	0.783	0.155	(−0.231, 0.542)	0.79	0.430
Model (12 to 24 months)	0.141	(−0.314, 0.596)	0.61	0.543	0.215	(−0.243, 0.672)	0.92	0.358
Model (24 to 36 months)	−0.001	(−0.611, 0.608)	−0.01	0.996	0.047	(−0.565, 0.658)	0.15	0.881
Model (36 to 48 months)	0.116	(−0.657, 0.888)	0.29	0.769	0.193	(−0.577, 0.964)	0.49	0.622
Height models								
Model (1 to 3 months)	−1.162	(−2.002, −0.321)	−2.71	0.007*	−0.685	(−1.473, 0.104)	−1.71	0.089
Model (3 to 6 months)	−0.562	(−1.474, 0.351)	−1.21	0.227	−0.426	(−1.316, 0.464)	−0.94	0.347
Model (6 to 12 months)	−0.109	(−1.054, 0.835)	−0.23	0.820	−0.021	(−0.941, 0.899)	−0.05	0.964
Model (12 to 24 months)	−0.024	(−1.162, 1.114)	−0.04	0.967	0.156	(−0.998, 1.310)	0.27	0.791
Model (24 to 36 months)	0.151	(−1.257, 1.559)	0.21	0.833	0.368	(−1.068, 1.803)	0.50	0.615
Model (36 to 48 months)	0.521	(−1.034, 2.077)	0.66	0.511	0.577	(−0.999, 2.153)	0.72	0.472

Interactions between covariates and month age were included in the adjustment models: (1) Weight models: maternal pre-pregnancy BMI (24 to 36 months), maternal pregnancy disease (36 to 48 months), child gender (1 to 3 months, 6 to 12 months), child birth weight (3 to 6 months, 6 to 12 months); (2) Height models: maternal pre-pregnancy BMI (1 to 3 months), maternal history of gestation (24 to 36 months), maternal age (6 to 12 months), child gender (1 to 3 months, 6 to 12 months), child birth weight (1 to 3 months, 36 to 48 months), exclusive breastfeeding from birth to 1 month (1 to 3 months, 12 to 24 months), child disease from birth to 1 month (1 to 3 months).

PPD, postpartum depression; CI, confidence interval.

* Level of significance: *P *< 0.05.

### 3.3. Association between postpartum EPDS score and children's physical growth

Before adjusting for confounders, associations were observed between postpartum EPDS scores and the weight and height growth rate of children aged 1–3 months (*β *= −0.024, 95% CI (−0.037, −0.012), *P* < 0.001; *β *= −0.071, 95% CI (−0.113, −0.029), *P* = 0.001) and 3–6 months (*β *= −0.021, 95% CI (−0.039, −0.004), *P* = 0.016; *β *= −0.047, 95% CI (−0.093, −0.002), *P* = 0.043) (Table 3). Additionally, the postpartum EPDS score was associated with the rate of child height gain (*β *= −0.082, 95% CI (−0.151, −0.013), *P* = 0.019) at 24–36 months old (Table 3).

**Table 3:** Association between postpartum EPDS score and children's physical growth at 1–48 months old.

Models	Unadjusted				Adjusted			
	*β*	95% CI	*t*	*P*-value	*β*	95% CI	*t*	*P*-value
Weight models								
Model (1 to 3 months)	−0.024	(−0.037, −0.012)	−3.80	<0.001*	−0.014	(−0.025, −0.002)	−2.26	0.024*
Model (3 to 6 months)	−0.021	(−0.039, −0.004)	−2.42	0.016*	−0.012	(−0.029, 0.004)	−1.47	0.143
Model (6 to 12 months)	−0.012	(−0.031, 0.008)	−1.20	0.232	−0.008	(−0.027, 0.012)	−0.77	0.444
Model (12 to 24 months)	−0.007	(−0.030, 0.015)	−0.63	0.530	−0.006	(−0.029, 0.017)	−0.49	0.628
Model (24 to 36 months)	−0.016	(−0.046, 0.014)	−1.04	0.301	−0.014	(−0.044, 0.017)	−0.87	0.385
Model (36 to 48 months)	−0.025	(−0.063, 0.014)	−1.25	0.211	−0.022	(−0.062, 0.017)	−1.12	0.262
Height models								
Model (1 to 3 months)	−0.071	(−0.113, −0.029)	−3.32	0.001*	−0.044	(−0.084, −0.004)	−2.17	0.030*
Model (3 to 6 months)	−0.047	(−0.093, −0.002)	−2.03	0.043*	−0.029	(−0.074, 0.016)	−1.29	0.199
Model (6 to 12 months)	−0.035	(−0.082, 0.011)	−1.48	0.138	−0.026	(−0.072, 0.021)	−1.09	0.277
Model (12 to 24 months)	−0.048	(−0.103, 0.008)	−1.68	0.093	−0.040	(−0.097, 0.017)	−1.37	0.171
Model (24 to 36 months)	−0.082	(−0.151, −0.013)	−2.35	0.019*	−0.069	(−0.141, 0.002)	−1.92	0.055
Model (36 to 48 months)	−0.067	(−0.144, 0.011)	−1.69	0.091	−0.054	(−0.135, 0.027)	−1.31	0.190

Interactions between covariates and month age which were included in the adjustment models were the same as that in the PPD models.

EPDS, edinburgh postpartum depression Scale; CI, confidence interval.

* Level of significance: *P* < 0.05.

In the adjusted models, postpartum EPDS scores were negatively associated with weight gain (*β *= −0.014, 95% CI (−0.025, −0.002), *P* = 0.024) and height growth (*β *= −0.044, 95% CI (−0.084, −0.004), *P* = 0.030) in children aged 1–3 months (Table 3) and no association between EPDS scores and child weight and height growth was observed in children aged 3–6 months, 6–12 months, 12–24 months, 24–36 months, and 36–48 months.

Moreover, in the double independent variable correction models, we found that children of pre-pregnancy thin mothers exhibited slower weight gain between 1 and 3 months (*β *= −0.190, 95% CI (−0.338, −0.041), *P* = 0.012; *β *= −0.189, 95% CI (−0.336, −0.042), *P* = 0.012) and 6 and 8 months (*β *= −0.324, 95% CI (−0.571, −0.077), *P* = 0.010; *β *= −0.307, 95% CI (−0.553, −0.062), *P* = 0.014) of age compared to those of overweight mothers, while children of pre-pregnancy thin mothers exhibited slower height growth between 24 and 36 months (*β *= −0.849, 95% CI (−1.547, −0.152), *P* = 0.017; *β *= −0.771, 95% CI (−1.4661, −0.075), *P* = 0.030) of age compared to those of normal weight mothers. Children of pre-pregnancy overweight mothers gained weight more rapidly between 12 and 24 months (*β *= 0.301, 95% CI (0.079, 0.523), *P* = 0.008; *β *= 0.300, 95% CI (0.078, 0.523), *P* = 0.008), 24 and 36 months (*β *= 0.364, 95% CI (0.063, 0.664), *P* = 0.018; *β *= 0.365, 95% CI (0.065, 0.665), *P* = 0.017), and 36 and 48 months (*β *= 0.601, 95% CI (0.217, 0.985), *P* = 0.002; *β *= 0.602, 95% CI (0.218, 0.985), *P* = 0.002) of age compared to those of normal-weight mothers. Children of mothers with at least a bachelor's degree grew faster in height between 6 and 12 months (*β *= 0.593, 95% CI (0.016, 1.170), *P* = 0.044; *β *= 0.588, 95% CI (0.012, 1.163), *P* = 0.045), 12 and 24 months (*β *= 0.852, 95% CI (0.148, 1.566), *P* = 0.018; *β *= 0.842, 95% CI (0.139, 1.545), *P* = 0.019), 24 and 36 months (*β *= 0.934, 95% CI (0.055, 1.813), *P* = 0.037; *β *= 0.941, 95% CI (0.066, 1.816), *P* = 0.035), and 36 and 48 months (*β *= 1.144, 95% CI (0.154, 2.134), *P* = 0.024; *β *= 1.153, 95% CI (0.165, 2.141), *P* = 0.022) of age compared to those of mothers with only senior high school education or below.

In addition, low birth weight children gained weight and length more slowly at 1–3 months (weight: *β *= −1.368, 95% CI (−1.616, −1.121), *P* < 0.001; *β *= −1.355, 95% CI (−1.601, −1.108), *P* < 0.001; height: *β *= −4.925, 95% CI (−5.826, −4.023), *P* < 0.001; *β *= −4.902, 95% CI (−5.802, −4.001), *P* < 0.001), 3–6 months (weight: *β *= −1.140, 95% CI (−1.5010, −0.779), *P* < 0.001; *β *= −1.110, 95% CI (−1.471, −0.750), *P* < 0.001; height: *β *= −1.963, 95% CI (−2.917, −1.010), *P* < 0.001; *β=*−1.945, 95% CI (−2.900, −0.992), *P* < 0.001), 6–12 months (weight: *β *= −0.759, 95% CI (−1.184, −0.334), *P* < 0.001; *β *= −0.733, 95% CI (−1.158, −0.308), *P* = 0.001; height: *β *= −1.502, 95% CI (−2.479, −0.524), *P* = 0.003; *β *= −1.458, 95% CI (−2.435, −0.481), *P* = 0.004), and 12–24 months (weight: (*β = *−0.548, 95% CI (−1.024, −0.071), *P* = 0.024; *β *= −0.520, 95% CI (−0.997, −0.044), *P* = 0.032; height: *β *= −1.314, 95% CI (−2.507, −0.122), *P* = 0.031; *β *= −1.228, 95% CI (−2.419, −0.036), *P* = 0.043) of age, and low birth weight children gained weight at a lower rate between 36 and 48 months (*β *= −0.942, 95% CI (−1.769, −0.115), *P* = 0.026; *β *= −0.889, 95% CI (−1.715, −0.063), *P* = 0.035) of age, compared to normal birth weight children. Furthermore, the growth rate of boys was higher than that of girls in each weight and length growth model from 1 to 48 months of age (*P* < 0.001).

## 4. Discussion

This birth cohort study is one of the first to explore the dynamic changes in the consequences of maternal PPD on the physical growth trajectory of children aged 1–48 months, based on LMM models. Our findings can assist in identifying and correcting the potential harm of mothers' PPD on children's physical growth as soon as possible. We did not find a dynamic association between PPD and weight and height growth in children aged 1–48 months in our cohort, although a negative association was observed in children aged 1–3 months old before adjusting for covariates. These findings are consistent with those of Ajslev et al. (6) and Grote et al. (11) from similar cohorts in Europe.

However, our analyses provide novel findings and add new evidence to the existing literature. We identified an association between maternal postpartum EPDS scores and children's physical growth that varied across different age groups. Although we found that maternal EPDS scores had a negative effect on children's weight and height growth rate at 1–3 months, there was no association at 3–6 months, 6–12 months, 12–24 months, 24–36 months, and 36–48 months old. Several factors may have contributed to this newly observed pattern.

First, the association between EPDS scores and physical development in infancy identified in our cohort may be related to difficulties parenting and impaired feeding behaviors in mothers with high EPDS scores. Previous studies have shown that mothers with postpartum depression are more likely to stop exclusive breastfeeding 1–3 months postpartum (39, 40); similarly, we found that 31.3% fewer mothers with PPD than without PPD exclusively breastfed their children in the first month postpartum, and that mothers who did not exclusively breastfeed their children reported PPD were 3.93 times likely than those who did exclusively breastfeed. After adjusting for covariates, we also found that a higher likelihood of mothers with PPD who were thinner before pregnancy and those who sicker during pregnancy, while a 12.4% higher percentage of mothers with PPD who low birth weight children before the adjustment, compared to non-postpartum depressed mothers. This suggests that PDD may have made it more difficult for mothers to exclusively breastfeed their children (40–42). Although no association was found between exclusive breastfeeding and the growth of children in any of the adjusted models, previous studies have reported that early cessation of exclusive breastfeeding is a major cause of malnutrition in children during infancy (43). The World Health organization recommends that mothers breastfeed their children up to 6 months after birth to ensure optimal growth and health, as breast milk is the optimal food for infants and contains a variety of immune substances and infection-fighting components (43).

Moreover, we found that children of pre-pregnancy thin mothers gained weight more slowly between 1 and 3 and between 6 and 12 months of age compared to those of overweight mothers and that low birth weight children gained weight and height more slowly between 1 and 24 months of age compared to normal birth weight children. In addition, mothers with lower monthly household incomes were more likely to report PPD, with mothers with a monthly household income of less than RMB 2,000 reporting 5.05 times more symptoms than mothers with a monthly household income of above RMB 2,000; this is consistent with the findings of a large community sample study conducted by Wu et al. in China (*n* = 300,000) (44). Thus, when mothers with PPD do not exclusively breastfeed their children and the mother's economic situation does not fully support bottle- or mixed-feeding, the physical growth of children with high nutritional needs during infancy might be slowed.

The first three months postpartum are also a time for mother–child pairs to adapt to feeding and being fed. Due to a loss of energy, a loss of interest in family activities and poor interaction abilities with their infants, mothers with PPD have difficulties caring for and feeding their infants (22, 23, 41). Previous research has shown that mothers with PPD are less likely to concentrate on their babies and more likely to force, restrict, and emotionally feed their children due to the clinical features of PPD (19, 20, 22, 23, 30, 39, 45). According to attachment theory, the more depressed mothers tend to force, restrict, and emotionally feed, the less likely their child is to develop a sense of security with the mother. This leads to an increased risk of children developing unhealthy eating behaviors, which can delay weight and height gain in children aged 1–3 months (19, 24).

A second possible explanation for our observations is that children of mothers with high EPDS scores begin to show catch-up growth at 3 months or earlier after birth. Previous studies have shown that mothers of low birth weight children are more likely to have PPD, and although low birth weight children grew more slowly after birth compared to children with a normal weight, adequate nutritional intake during infancy can lead to catch-up growth (45, 46). Although we found, before adjusting for confounders, that mothers of low birth weight children were more likely to report PPD and that children of mothers reporting PPD had a lower weight and height over the entire study period than those of mothers without symptoms, we also found gradual increases in weight and height growth rates at 1–3, 3–6, 6–12, and 12–24 months in low birth weight children (after adjusting for confounders); there was, however, no difference in rates between 24 and 36 months of age.

Third, the duration of PPD may play a mediating role in the changing association between EPDS scores and child weight and height growth. A 2020 edition of the code for the diagnosis and treatment of mental disorders reported that the average duration of a single depressive episode is about 16 weeks, while if not treated, it can last for 6 months or more (47). Lu et al. reported that patients with depression in China rarely received adequate treatment, with only 4.7% of them seeking psychotherapy (48). Moreover, previous studies have shown that mothers can experience depression for long periods of time, from 2 weeks to 6 months after delivery, which reflects that PPD may become chronic (9, 49). The association between postpartum EPDS scores and children's physical growth after 1 month postpartum requires additional investigation (50). We recommended mothers who reported PPD to seek further assistance from mental health consulting clinics. However, due to the specific focus of this study and time constraints, treatment outcomes and symptom duration were not collected. Therefore, we were unable to examine the association between the duration of PPD and children's physical growth. Future studies should be conducted to further clarify the role of PPD duration and its long-term effects on children's physical growth.

In addition, we found that maternal pre-pregnancy BMI, education level, and child sex contributed most to differences in child weight and height gain after infancy. Although we did not find a difference in the reported rate of PPD between pre-pregnancy overweight and normal weight mothers, children of pre-pregnancy overweight mothers gained weight more rapidly and at an increasing rate compared to those of normal weight mothers. We also found that the likelihood of reporting PPD was 3.32 times higher in mothers who were thin before pregnancy than in those of normal weight. Similarly, although we did not find differences in the rate of reporting PPD among between with different levels of education, previous studies have shown that maternal education was negatively associated with maternal postpartum depression (44). We also found that children of mothers with higher levels of education grew faster in height, and that growth rates increased with age. This suggests that a healthy pre-pregnancy weight of the mother and a good family education environment contribute to the psychological health of the mother and the growth of the child after delivery.

Our findings emphasize the importance of promoting positive feeding and parenting practices among depressed mothers to stimulate their children's growth and development as soon as possible. As depressed mothers are often unable to exclusively breastfeed their children and struggle with their mental health, more family and social support is needed to help them provide adequate care to their children, who are highly dependent on them in the early stages of their life, especially during the first 3 months postpartum.

### 4.1. Limitations

This study had some limitations. First, we followed participants up for only 4 years, and lost a significant percentage of the original sample population. Participants moving to a new residence or changing their contact information were the main reasons for the loss of follow-up in this study. In addition, another reason was that some participants declined to continue due to the frequent appointments put a burden on them. Although the loss and analysis samples were comparable in baseline data, we may have underestimated the effects of maternal PPD on the physical growth of children, owing to the overall low rate of PPD in our community sample. Only 3.3% of mothers reported symptoms of PPD at 1 month postpartum, which is much lower than what previous studies have reported (7%–25%) (1–3). One possible reason for this might be that Chinese mothers are reluctant to report their PPD accurately, as they are expected to see childbirth as a reason for celebration in Chinese culture (51). Another potential explanation for the lower-than-expected rate may be that depressed mothers are less likely to participate in studies due to their low energy levels and their lack of interest in social activities (22, 23, 41). Second, while we excluded mothers with severe neuropsychiatric or organic diseases and collected information about illnesses and treatment during pregnancy, we did not control for the effect of prenatal depression and did not assess the duration and changes in PPD. Future studies are needed to measure depression of mothers at different time points before and after childbirth to more accurately determine the association between PPD and children's growth and development.

## 5. Conclusions

While we did not find a sustained and dynamic association between maternal PPD and children's weight and height growth, postpartum EPDS scores were negatively associated with the weight and height growth rate of children in our community sample; the association varied with age but was only significant in the first 3 months of life. Generally, this study aims to improve the understanding of the dynamic effects of untreated maternal postpartum depression on the physical growth of children, which can help guide appropriate strategies for identifying maternal depressive symptoms in time and promoting appropriate child development.

## Data Availability

The datasets presented in this article are not readily available due to the privacy of research participants. Requests to access the datasets should be directed to YY, yanyan@csu.edu.cn.
